# Successful Treatment With Single-Fraction Stereotactic Body Radiotherapy to Lung and Reirradiation of Neck Node Metastases in a Treated Base Tongue Primary

**DOI:** 10.1155/crom/1608574

**Published:** 2025-09-23

**Authors:** Aakriti Bhardwaj, Sindhu Tanigassalam, Shambhavi C., Shirley Lewis

**Affiliations:** ^1^Department of Radiotherapy and Oncology, Kasturba Medical College, Manipal, Manipal Academy of Higher Education, Manipal, Karnataka, India; ^2^Department of Nuclear Medicine, Kasturba Medical College, Manipal, Manipal Academy of Higher Education, Manipal, Karnataka, India; ^3^Department of Radiotherapy and Oncology, Manipal College of Health Professionals, Manipal Academy of Higher Education, Manipal, Karnataka, India

**Keywords:** head and neck cancers, nodal recurrence, oligometastatic disease, reirradiation, stereotactic body radiotherapy

## Abstract

**Introduction:** Head and neck cancers are one of the most common cancers globally. Locoregional recurrence or distant metastasis is a common mode of recurrence and should be treated curatively, where feasible. We present a case of an elderly gentleman treated with reirradiation for nodal metastases and lung metastases ablated with single-fraction SBRT.

**Case Summary:** An elderly 73-year-old gentleman treated with definitive radiotherapy 3 years ago for carcinoma of the base of the tongue presented with the complaint of left neck swelling for 3 weeks. A 2 × 3 cm left Level II lymph node was palpable with skin involvement. The positron emission tomography (PET) was suggestive of a left Level II cervical node measuring 4 × 4 × 3.2 cm, encasing the carotid artery and involving the skin, and a 3 × 2 cm right lower lobe lung lesion, suggestive of a metachronous lung primary or metastasis. The neck node was deemed unresectable due to more than 180-degree contact with vessels. The patient was unwilling to undergo a biopsy of the lung. Given the age, performance status and oligometastatic presentation, the patient was planned for radical intent treatment to both the neck node and the lung lesion. He was treated with reirradiation to the neck node with a dose of 66 Gy in 33 fractions over 6.5 weeks and SBRT 34 Gy in a single fraction to the lung lesion. He developed neck soft tissue necrosis, at 1.5 years after radiation, which resolved on antibiotics. The patient remains clinically well, and the PET scan performed at 1 and 2 years following radiation showed complete response with no new metastasis.

**Conclusion:** Radical intent can be considered in oligometastatic head and neck cancers. Reirradiation is a treatment option for patients with recurrent head and neck cancers. Single-fraction SBRT is one of the recommended dose schedules for lung metastases.

## 1. Introduction

Head and neck cancers (HNC) are one of the common cancers globally, with an age-standardised incidence of 5.8% [1]. In India, cancer of the lip and oral cavity ranks first amongst men and second amongst both sexes [2]. HNC accounts for 26% of cases amongst males and 8% amongst females [3]. The burden in India is higher than in developed nations and peaks in the age group above 60 years. The incidence of carcinoma of the oropharynx, as per GLOBOCAN data, is 44.2% in Asia [2]. However, in India, the oral cavity is the leading site, followed by the larynx, hypopharynx and tonsil [3]. Definitive chemoradiation is the standard of care for locally advanced oropharyngeal cancer, resulting in 5-year survival of over 75% overall and over 90% in human papillomavirus (HPV)–positive cases [4].

Despite the good prognosis, a majority of patients relapse within 5 years of treatment. Amongst HPV-positive patients, around 25% relapse by 5 years. A large recurrence pattern study amongst HNCs from Michigan (*n* = 447) showed that around 85% relapse within 2 years, with an average time to relapse of 12 months. Locoregional relapse was more common, followed by distant and synchronous locoregional, and distant. Haematogenous spread in HNC ranges from 1.6% to 23%, with the lungs being the most common site of metastases, followed by the bone and liver [5]. HPV positivity resulted in delayed recurrence, distant-only metastases and better survival with salvage therapies [6].

Treatment of recurrent HNC is challenging. Isolated locoregional recurrence may be managed with surgery or reirradiation [7, 8]. Reirradiation can provide effective local control in selective cases [8]. Isolated distant metastases or oligometastatic disease (OMD) can be considered for surgery or ablation with stereotactic body radiotherapy (SBRT), whilst widely metastatic disease requires systemic therapy with immunotherapy and/or chemotherapy [9]. Haring et al. showed that oligometastatic patients treated with surgery or SBRT had a better survival compared to the rest of the cohort (29.3 months vs. 10.4 months, *p* < 0.001) [6]. An international multi-institutional consortium results by Id Said et al. showed good local control and median survival of 23 months in OMD (< 5 metastases) treated with SBRT [10]. The series of pulmonary OMD treated with SBRT shows excellent local control and 2-year survival of 35%–61% [11, 12]. These suggest that local radiotherapy with SBRT or reirradiation has the potential to provide good local control whilst deferring systemic therapy, which is very valuable in preserving the quality of life of elderly patients.

We present a case of an elderly gentleman diagnosed with HPV-negative carcinoma of the base of the tongue (cT2N0M0 previously treated with radical radiation) with synchronous neck node recurrence and lung metastases after a disease-free interval of 4 years. We successfully treated the neck node recurrence with reirradiation to 66 Gy and the lung with SBRT 34 Gy in a single fraction. The patient has completed a 2-year follow-up with a complete response at both sites. This case exemplifies that selective radical radiotherapy treatment is effective in oligometastatic cancers in an elderly patient, offering efficient disease control, warding off the need for systemic therapy.

## 2. Case Summary

**Figure 1:**
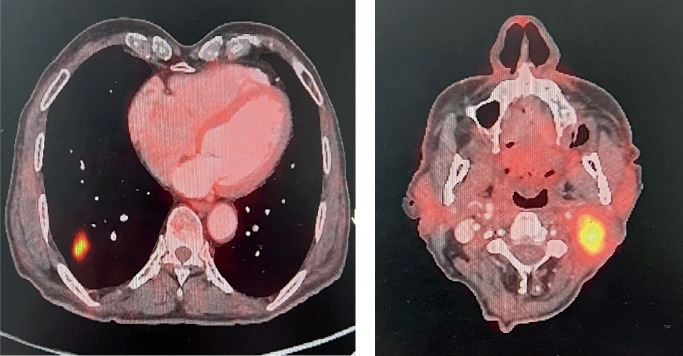
Positron emission tomography scan images. (a) Right lung lower lobe lesion measuring 2 × 3 cm. (b) 4 × 4 × 3.2 cm left cervical node with vessel and skin involvement.

A 73-year-old gentleman presented with left neck swelling for 2–3 weeks. He was treated with definitive radiotherapy 3 years back for HPV-negative carcinoma of the base of the tongue cT2N0M0 to a dose of 66 Gy in 30 fractions with volumetric modulated arc therapy (VMAT). On local examination, a 2 × 3 cm left Level II lymph node was palpable, which was hard in consistency, partly mobile, and the skin was not pinchable. A thorough oral cavity examination did not show any evidence of growth. A positron emission tomography–computed tomography (PET-CT) was done, suggesting a 4 × 4 × 3.2 cm left cervical node with vessel involvement and skin involvement with a standardised uptake value (SUV) max of 9.15 (Figure 1). Another lung lesion measuring 2 × 3 cm in the right lower lobe with SUV max of 8.11 was identified with no other metastases. The patient underwent fine needle aspiration cytology (FNAC) from the left neck node, which was suggestive of squamous cell carcinoma. The patient was offered a biopsy of the lung lesion to differentiate metastases from primary. The patient and his family were reluctant due to his age. The single lung lesion could be a new primary or metastasis, but given the PET avidity and synchronous neck node lesion, we deemed it to be an oligometastases. The case was discussed in a multidisciplinary tumour board, and since the neck node had > 180 contact with vessels, achieving R0 was unlikely; systemic therapy was discussed with the patient and family, but they were unwilling, fearing the side effects. The patient was hence planned for radical reirradiation to the neck and SBRT to the lung lesion.

**Figure 2:**
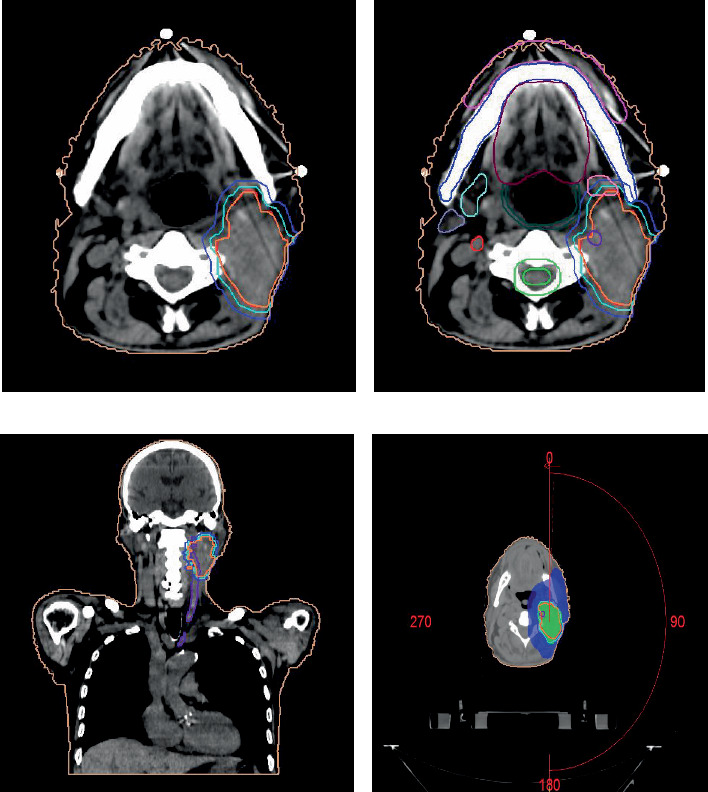
Contouring volumes on computed tomography scan for neck node. (a) Axial section: GTV node in bright red: 66 Gy, CTV in cyan: 59.4 Gy, PTV in purple: 59.4 Gy. (b) Axial section: lips: pink, mandible: dark blue, right parotid: mauve, right submandibular gland: light blue, oral cavity: maroon, right carotid artery: red, spinal cord: green, spinal cord planning risk volume (PRV): light green, left submandibular gland: light pink, left carotid artery: dark purple. (c) Coronal section showing GTV encasing left carotid artery. (d) Axial section showing dose coverage of the GTV.

He was treated for the neck node using VMAT to a dose of 66 Gy in 33 fractions over 6.5 weeks with curative intent. He underwent a radiotherapy planning computed tomography (CT) scan in a supine position with a head and neck thermoplastic mould. The gross tumour volume (GTV) included the left cervical lymph node, and the high-risk clinical target volume (CTV) included the GTV + 3 mm margin around it. The planned dose was 66 Gy in 33 fractions for GTV (EQD2 119.1 Gy) and 54 Gy in 33 fractions to high-risk CTV (Figure 2). The dose constraints for all organs were met (Table 1), as per Garg et al. [13]. The spinal cord dose was EQD2 65.4 Gy. He was treated with a volumetric modulated arc therapy technique using 6 MV photons over 5.5 weeks, 5 days per week.

**Figure 3:**
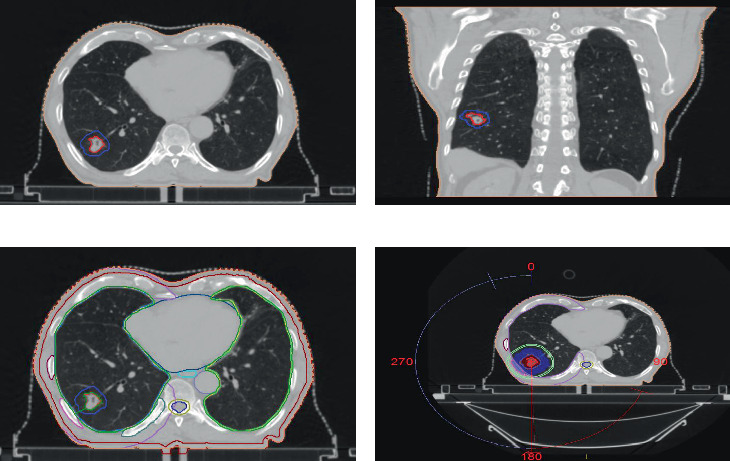
Contouring volumes on computed tomography scan for lung lesion. (a) Axial section: GTV in bright red: 34 Gy, PTV in dark blue: 34 Gy. (b) Coronal section: GTV and PTV. (c) Axial section: skin: maroon, right eight rib: dark red, right ninth rib: pink, chest wall: light purple, heart: green, oesophagus: cyan, spinal cord: dark blue, spinal cord PRV: muddy green, ipsilateral lung: green, contralateral lung: light green. (d) Axial section showing dose coverage of the GTV, dose wash within 2 cm ring.

The lung nodule was treated using the SBRT technique with a dose of 34 Gy in a single fraction. He underwent a radiotherapy planning CT scan in a supine position with a thoracic thermoplastic mould using the automated breath coordinator (ABC) technique in deep inspiratory breath hold (DIBH). The GTV included the lung lesion, and a 5 mm planning target volume (PTV) was generated. The planned dose was 34 Gy in one fraction (Figure 3). The dose constraints for all organs were met as per RTOG 0915 (Table 2) [14]. He was treated with the SBRT technique using 6 MV FFF energy in a single fraction.

**Figure 4:**
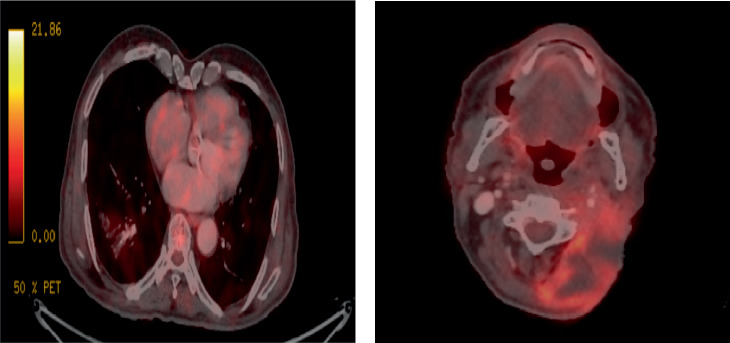
Positron emission tomography scan images of follow-up at second year. (a) Right lung lower lobe lesion showing complete response. (b) Abscess in the left neck.

Follow-up: Patient was followed up weekly whilst receiving radiation. He developed Grade I dermatitis, Grade I xerostomia and Grade I dysphagia during the course of treatment. He tolerated the treatment well. At 3 months, there was a complete clinical response, and PET-CT showed a complete response in the neck node and lung with no new metastases. Patient has completed 2 years of follow-up with a sustained complete response in the neck node. Patient is doing well and is able to carry out his daily activities with minimal difficulty, with only Grade I xerostomia and Grade I lung radiological changes on scans. The PET-CT showed a complete response at both sites in 1 year. He remained on a 3-month follow-up. At 2 years, he presented with left neck erythema, pain and fever. The PET-CT was suggestive of an abscess in the neck with no metastasis in the lung (Figure 4). A biopsy was taken from the neck lesion, and it was negative for malignancy. He was managed conservatively with prolonged antibiotics as the pus culture showed infection. The ulcer, which developed over the left neck Level II after infection, is healing. This presentation could be due to soft tissue necrosis, a late sequelae of reirradiation.

## 3. Discussion

Locoregional recurrences are common and can affect as many as 24%–50% of patients. Haring et al. show that patients with HPV-positive disease were more likely to have distant metastases to the lung. In contrast, patients with HPV-negative disease were more likely to have locoregional recurrence [6]. As per Curtis et al., the use of reirradiation is beneficial for locoregional recurrence or new primary in HNC patients as a salvage treatment approach despite its known risk of toxicities [15]. The study shows a trend towards improved overall survival (OS) amongst postoperative reirradiation following salvage surgery patients. Reirradiation is often feasible based on time from prior radiation, patient performance status and existing toxicities. Reirradiation of recurrence or second primary squamous carcinoma appears safe in the modern era with an improved therapeutic ratio [8]. Patients more than 2 years from their previous treatment with resectable tumours have better long-term survival [8]. Reirradiation should be considered a treatment option for patients with recurrent HNC.

Reirradiation requires meticulous planning and consideration of the prior radiation doses with strict constraints for the spinal cord, carotid, bone and mucosa. A dreaded and often lethal complication of reirradiation for HNC and/or HNC therapy is carotid blowout syndrome, in which the carotid artery or one of its major branches ruptures. Known risk factors of carotid blowout syndrome are tumour invasion of the carotid arteries, infection, surgery and high cumulative doses of radiotherapy. Another side effect that can cause considerable suffering for the patient is osteoradionecrosis (ORN). Hence, careful selection is the key. Apart from ORN and carotid artery blowout, another long-term toxicity of head and neck reirradiation is soft tissue necrosis. It is a severe late complication resulting from cumulative radiation-induced vascular damage and impaired tissue healing. It presents as progressive ulceration, fibrosis and tissue breakdown. It is often accompanied by pain, infection and fistula formation. Bots et al. reported long-term disease control and late radiation toxicity for patients reirradiated to a dose of 45 Gy for HNC [16]. Out of 137 patients, only two developed Grade 4 soft tissue necrosis.  Table 3 depicts a summary of studies with post–head and neck reirradiation, showing the number of patients with toxicities and dose predictors of toxicity after reirradiation, and the cumulative maximum dose [17]. Soft tissue necrosis was seen in our patient at one and a half years and managed conservatively.

SBRT is a commonly employed technique to treat unresectable patients with early stage nonsmall cell lung cancer (NSCLC). GORTEC 2014-04 Phase 2 study assesses survival without definitive quality of life deterioration of omitting upfront chemotherapy in oligometastatic patients with HNSCC using SABR alone [18]. Eligible participants were randomly assigned (1:1) to receive chemo-SABR or SABR alone. It was seen that the SBRT-alone arm resulted in similar survival but better quality of life and fewer toxicities. Thus, it was inferred that SABR alone could be considered in oligometastatic patients with HNSCC [18]. Franzese et al. compared SBRT and palliative radiotherapy in oligometastatic salivary gland cancers and reported superior local control at 1 year with SBRT [19]. Schulz et al. showed that metastasis-directed treatment, SBRT or surgery, had a three times better impact in terms of median survival than untreated patients with a potentially treatable disease [20]. In a long-term analysis of SABR-COMET, the effects of SBRT on OS showed a median OS benefit of 22 months [9]. Out of a total of 191 metastatic lesions, 55 were lung metastases. SABR did not result in a detriment in QOL. It also benefitted patients with a controlled primary tumour and 1–5 oligometastases [9].

RTOG 0915 was a randomised Phase II trial of SBRT for unresectable peripheral early stage NSCLC patients that compared 34 Gy in a single fraction to 48 Gy in four fractions, with 1-year primary endpoint being Grade 3 or higher toxicities. The single-fraction schedule showed a lower rate of toxicities for comparable primary tumour control [13]. Trans-Tasman Radiation Oncology Group (TROG) 13.01/Australasian Lung Cancer Trials Group (ALTG) 13.001 trial, in the final updated analysis, showed that there was no evidence that OS was associated with fractionation [21]. Single-fraction SBRT is one of the recommended dosing schedules for both lung primary and metastases. In the present case report, single-fraction schedule was convenient and well tolerated.

One of the limitations was that we treated the lung lesion without biopsy. It is often difficult to obtain a biopsy for a lung primary, especially in elderly patients. Koh et al. showed no difference in outcome for early stage lung cancer patients treated with SBRT, irrespective of biopsy confirmed or radiologically detected [22]. However, in the present case, it would be prudent to undergo a biopsy to differentiate between metastases and a lung primary. SBRT for reirradiation for recurrent HNC is increasingly being used [23]. Delerue et al. studied SBRT (36 Gy in six fractions) in a large retrospective cohort (*n* = 110) of recurrent HNCs [24]. The median survival was 20.8 months, with 42% Grade 2 or higher toxicity at 2 years. Yamazaki et al. report outcomes of 46 patients with isolated nodal recurrence from HNC treated with SBRT (32 Gy in five fractions) [25]. One-year local control was 49% with Grade 3 or higher toxicity of 8.9%. All were Grade 5 carotid blowout related to tumour invasion or high dose to the carotids. The authors suggest small nodes with a volume of less than 25 cc are suitable for SBRT. In the present case, the node was extensive, with skin and vessel involvement; therefore, we favoured conventional reirradiation.

## 4. Conclusion

Radical intent can be considered in oligometastatic cancers. Reirradiation is a treatment option for patients with recurrent HNCs. Single-fraction SBRT is a recommended dosing schedule for lung primaries and metastases.

## 5. Patient Perspective

I noticed a neck swelling, and we went to the ENT department. I expected it to be a cold infection. However, when the doctor asked to get the fine needle biopsy done, it was evident that the cancer was back. However, we as a family still hoped to see better results. The result confirmed the return of cancer, and the PET-CT confirmed that it is extending not only to the skin tissue but also to a second lesion in the right lung. This is kind of a shock to us. Further discussion with the oncologists confirmed that the node is extending to the skin, and surgery is not an option for the neck. So there were two options to go with: radiation and chemotherapy or only radiation. After discussing with the family, we decided that the quality of life is more important than extending years. So putting myself through the chemo with its side effects is not worth the risk. We decided to go with the radiation.

We were aware of the radiation to the neck, but the radiation to the lung lesion was new to us. It was a bit difficult for me to go through the radiation to the lung. It was a single-time process. The neck radiation was much smoother. We had taken the preferred slot to visit the hospital, and it was easy to manage. This time around, I had fewer symptoms and was able to take food without much difficulty.

Postradiation and the follow-up visits are not too tiresome, and we are now getting used to them. The recent PET scan was clean, too, and we hope this will remain so for a long time.

## Figures and Tables

**Table 1:** Doses achieved and constraints for neck reirradiation.

**Description**	**Dose achieved**	**Dose constraint**
Spinal cord	34.98 Gy	Dmax < 35 Gy

Contralateral carotid (right)	Dmean 5.45 Gy	Dmean < 60 Gy
	13 Gy	0.1 cc < 106 Gy
	11.96 Gy	1 cc < 99 Gy
	0 cc	V70 < 2 cc
	0 cc	V100 < 1 cc
	0 cc	V120 < 0 cc

Ipsilateral carotid (left)	Dmean 27.53 Gy	Dmean < 60 Gy
	66.37 Gy	0.1 cc < 106 Gy
	65.36 Gy	1 cc < 99 Gy
	0 cc	V70 < 2 cc
	0 cc	V100 < 1 cc
	0 cc	V120 < 0 cc

Constrictors	25.32 Gy	Dmean < 73 Gy

Brainstem	Dmax 18 Gy	0.1 cc < 50 Gy

Mandible	Dmax 64 Gy	0.1 cc < 101 Gy

Right parotid	Dmean 7 Gy	Dmean < 37.5 Gy

Left parotid	Dmean 39.37 Gy	Dmean < 37.5 Gy

Larynx	Dmean 4.34 Gy	Dmean < 44 Gy

Thyroid	Dmean 0.36 Gy	—

Right submandibular gland	Dmean 13 Gy	—

Left submandibular gland	Dmean 55 Gy	—

Oesophagus	Dmean 0.24 Gy	—

**Table 2:** Doses achieved and constraints for stereotactic body radiotherapy to lung 34 Gy/#1.

**Description**	**Dose constraint achieved**	**Dose constraint**
PTV 34 Gy	34.84 Gy	D95 > 34 Gy
	33.4 Gy	D99 > 30.6 Gy
	46.39 Gy	D0cc < 42.5 Gy

Aorta	3.97 Gy	D0cc < 37 Gy
	2.07 Gy	D10cc < 31 Gy

Bronchus	0.4 Gy	D0cc < 20 Gy
	0.16 Gy	D4cc < 10.5 Gy

Oesophagus	2.6 Gy	D0cc < 15.4 Gy
	1.19 Gy	D5cc < 11.9 Gy

Heart	16 Gy	D0cc < 22 Gy
	6.22 Gy	D15cc < 16 Gy

Lungs-GTV	1.31 Gy	Dmean < 12 Gy
	0.92%	V20 < 10.15%

Ribs	15.13 Gy (10th rib), 27.4 Gy (9th rib), 17.6 Gy (8th rib)	D0cc < 30 Gy

Trachea	10.7 Gy	D0cc < 20 Gy
	5.2 Gy	D4cc < 10.5 Gy

Spinal cord	4.79 Gy	D0cc < 14 Gy

Spinal cord, PRV	4.97 Gy	D0cc < 15 Gy

Chest wall	25.66 Gy	D0.1 cc < 30 Gy

Skin	13.11 Gy	D0.1 cc < 26 Gy
	8.62 Gy	D10 cc < 23 Gy

**Table 3:** Summary of studies with post–head and neck reirradiation.

**Study**	**Technique and dose fractionation**	**Carotid blowout (** **n** **)**	**Carotid blowout (cumulative maximum dose)**	**Osteoradionecrosis ** **(** **n** **)**	**Osteoradionecrosis (cumulative maximum dose)**	**Soft tissue necrosis ** **(** **n** **)**
Bots et al.	IMRT, 60-66 Gy to primary and 50Gy to elective neck	1	—	8	114 Gy	2
Garg et al.	IMRT (72%) 3DCRT (28%) Median dose 60 Gy	1	120 Gy	—	—	—
Embring et al.	IMRT (93%) Median dose 59 Gy	2	119 Gy	2	119 Gy	—

## Data Availability

The data is available on request from the corresponding author.

## Nomenclature

SBRT: stereotactic body radiotherapy
HNC: head and neck cancer
HPV: human papilloma virus
OMD: oligometastatic disease
SUV: standardised uptake value
PET: positron emission tomography
VMAT: volumetric modulated arc therapy
FNAC: fine needle aspiration cytology
SCC: squamous cell carcinoma
ABC: automated breath coordinator
DIBH: deep inspiratory breath hold
GTV: gross tumour volume
CTV: clinical target volume
PTV: planning target volume
ORN: osteoradionecrosis
OAR: organs at risk
RTOG: Radiotherapy Oncology Group
NSCLC: nonsmall cell lung cancer
HR: hazard ratio
PFS: progression-free survival

## References

[1] Bray F., Laversanne M., Sung H. (2024). Global Cancer Statistics 2022: GLOBOCAN Estimates of Incidence and Mortality Worldwide for 36 Cancers in 185 Countries. *CA: A Cancer Journal for Clinicians*.

[2] Ferlay J., Colombet M., Soerjomataram I. (2021). Cancer Statistics for the Year 2020: An Overview. *International Journal of Cancer*.

[3] Bagal S., Budukh A., Thakur J. S. (2023). Head and Neck Cancer Burden in India: An Analysis From Published Data of 37 Population-Based Cancer Registries. *Ecancermedicalscience*.

[4] De Felice F., Bird T., Michaelidou A. (2020). Radical (Chemo) Radiotherapy in Oropharyngeal Squamous Cell Carcinoma: Comparison of TNM 7th and 8th Staging Systems. *Radiotherapy and Oncology*.

[5] Wedman J., Balm A. J., Hart A. A. (1996). Value of Resection of Pulmonary Metastases in Head and Neck Cancer Patients. *Head & Neck*.

[6] Haring C. T., Kana L. A., Dermody S. M. (2023). Patterns of Recurrence in Head and Neck Squamous Cell Carcinoma to Inform Personalized Surveillance Protocols. *Cancer*.

[7] Goodwin W. J. (2000). Salvage Surgery for Patients With Recurrent Squamous Cell Carcinoma of the Upper Aerodigestive Tract: When Do the Ends Justify the Means?. *Laryngoscope*.

[8] Ward M. C., Riaz N., Caudell J. J. (2018). Refining Patient Selection for Reirradiation of Head and Neck Squamous Carcinoma in the IMRT Era: A Multi-Institution Cohort Study by the MIRI Collaborative. *International Journal of Radiation Oncology • Biology • Physics*.

[9] Palma D. A., Olson R., Harrow S. (2020). Stereotactic Ablative Radiotherapy for the Comprehensive Treatment of Oligometastatic Cancers: Long-Term Results of the SABR-COMET Phase II Randomized Trial. *Journal of Clinical Oncology*.

[10] Id Said B., Mutsaers A., Chen H. (2023). Outcomes for Oligometastatic Head and Neck Cancer Treated With Stereotactic Body Radiotherapy: Results From an International Multi-Institutional Consortium. *Head & Neck*.

[11] Bonomo P., Greto D., Desideri I. (2019). Clinical Outcome of Stereotactic Body Radiotherapy for Lung-Only Oligometastatic Head and Neck Squamous Cell Carcinoma: Is the Deferral of Systemic Therapy a Potential Goal?. *Oral Oncology*.

[12] Pasalic D., Betancourt-Cuellar S. L., Taku N. (2020). Outcomes and Toxicities Following Stereotactic Ablative Radiotherapy for Pulmonary Metastases in Patients With Primary Head and Neck Cancer. *Head & Neck*.

[13] Videtic G. M., Hu C., Singh A. K. (2015). A Randomized Phase 2 Study Comparing 2 Stereotactic Body Radiation Therapy Schedules for Medically Inoperable Patients With Stage I Peripheral Non-Small Cell Lung Cancer: NRG Oncology RTOG 0915 (NCCTG N0927). *International Journal of Radiation Oncology • Biology • Physics*.

[14] Garg S., Kilburn J. M., Lucas J. T. (2016). Reirradiation for Second Primary or Recurrent Cancers of the Head and Neck: Dosimetric and Outcome Analysis. *Head & Neck*.

[15] Curtis K. K., Ross H. J., Garrett A. L. (2016). Outcomes of Patients With Loco-Regionally Recurrent or New Primary Squamous Cell Carcinomas of the Head and Neck Treated With Curative Intent Reirradiation at Mayo Clinic. *Radiation Oncology*.

[16] Bots W. T. C., van den Bosch S., Zwijnenburg E. M. (2017). Reirradiation of Head and Neck Cancer: Long-Term Disease Control and Toxicity. *Head & Neck*.

[17] Embring A., Onjukka E., Mercke C. (2021). Re-Irradiation for Head and Neck Cancer: Cumulative Dose to Organs at Risk and Late Side Effects. *Cancers*.

[18] Thariat J., Bosset M., Falcoz A. (2025). Survival Without Quality of Life Deterioration in the GORTEC 2014-04 “OMET” Randomized Phase 2 Trial in Patients with Head and Neck Cancer With Oligometastases Using Stereotactic Ablative Radiation Therapy (SABR) Alone or Chemotherapy and SABR. *International Journal of Radiation Oncology∗ Biology∗ Physics*.

[19] Franzese C., Ingargiola R., Tomatis S. (2022). Metastatic Salivary Gland Carcinoma: A Role for Stereotactic Body Radiation Therapy? A Study of AIRO-Head and Neck Working Group. *Oral Diseases*.

[20] Schulz D., Wirth M., Piontek G. (2018). Improved Overall Survival in Head and Neck Cancer Patients After Specific Therapy of Distant Metastases. *European Archives of Oto-Rhino-Laryngology*.

[21] Siva S., Sakyanun P., Mai T. (2023). Long-Term Outcomes of TROG 13.01 SAFRON II Randomized Trial of Single-Versus Multifraction Stereotactic Ablative Body Radiotherapy for Pulmonary Oligometastases. *Journal of Clinical Oncology*.

[22] Koh T. L., Ong W. L., Farrugia B., Leong T., Lapuz C., Lim A. (2022). To Biopsy or Not to Biopsy? Outcomes Following Stereotactic Body Radiotherapy (SBRT) for Biopsy-Confirmed Versus Radiologically-Diagnosed Primary Lung Cancer in a Single Australian Institution. *Asia-Pacific Journal of Clinical Oncology*.

[23] Mohamad I., Karam I., El-Sehemy A. (2023). The Evolving Role of Stereotactic Body Radiation Therapy for Head and Neck Cancer: Where Do We Stand?. *Cancers*.

[24] Delerue C., Pasquier D., Bogart E. (2024). Stereotactic Reirradiation in the Treatment of Head and Neck Cancers: A Retrospective Study on the Long-Term Experience of the Oscar Lambret Center. *Radiotherapy and Oncology*.

[25] Yamazaki H., Suzuki G., Aibe N. (2024). Re-Irradiation for Isolated Neck Recurrence in Head and Neck Tumor: Impact of rN Category. *Scientific Reports*.

